# Drugging a Stem Cell Compartment Using Wnt3a Protein as a Therapeutic

**DOI:** 10.1371/journal.pone.0083650

**Published:** 2014-01-06

**Authors:** Girija R. Dhamdhere, Mark Y. Fang, Jie Jiang, Katherine Lee, Du Cheng, Rebecca C. Olveda, Bo Liu, Kimberley A. Mulligan, Jeffery C. Carlson, Ryan C. Ransom, William I. Weis, Jill A. Helms

**Affiliations:** 1 Division of Plastic and Reconstructive Surgery, Department of Surgery, Stanford School of Medicine, Stanford, California, United States of America; 2 Department of Developmental Biology, Howard Hughes Medical Institute (HHMI), Institute for Stem Cell Biology and Regenerative Medicine, School of Medicine, Stanford University, Stanford, California, United States of America; 3 Departments of Structural Biology and Molecular and Cellular Physiology, Stanford School of Medicine, Stanford, California, United States of America; Simon Fraser University, Canada

## Abstract

The therapeutic potential of Wnt proteins has long been recognized but challenges associated with in vivo stability and delivery have hindered their development as drug candidates. By exploiting the hydrophobic nature of the protein we provide evidence that exogenous Wnt3a can be delivered in vivo if it is associated with a lipid vesicle. Recombinant Wnt3a associates with the external surface of the lipid membrane; this association stabilizes the protein and leads to prolonged activation of the Wnt pathway in primary cells. We demonstrate the consequences of Wnt pathway activation in vivo using a bone marrow engraftment assay. These data provide validation for the development of WNT3A as a therapeutic protein.

## Introduction

Therapeutic proteins are delivered to cells to supplement or replace inadequate or dysfunctional proteins, and are widely considered to be a direct and safe approach for the treatment of human diseases. Therapeutic proteins offer a distinct advantage over small molecules because of their specificity in mechanism of action and their potency, but they are limited by three factors: the stability, delivery and immunogenicity of the protein. Several strategies have been employed to address these limitations, including packaging the protein in flexible lipid formulations [1]. These lipid formulations can be extensively modified by changing the surface charge, the surface hydrophobicity, and the fluidity of the membrane, which in turn modulate the in vivo stability and release rates of the therapeutic protein [2].

Wnts are secreted, lipid modified [3], [4] glycoproteins and are viable candidates for therapeutic proteins. In addition to their well characterized roles in embryonic development and tissue homeostasis, Wnts also play an essential role in injury repair: the act of injury triggers activation of the endogenous Wnt pathway at or near the site of damage (reviewed in [5]), and this endogenous Wnt stimulus is subsequently required for the repair and/or regeneration of the injured tissue ([6]–[10] and reviewed in [11]).

The mechanism of Wnt action during the healing process has become increasingly clear: Wnts are potent stem cell-inducing growth factors that promote the proliferation and self-renewal of endogenous stem cells, which contribute to tissue repair [11] [12], [13]. Purification of Wnt3a [3] enabled initial development of Wnts as a therapeutic but the hydrophobic nature of these proteins precluded their in vivo use [14].

Here, we report on our development of Wnt3a as a therapeutic protein. In previous work we showed that liposomal packaging preserves the biological activity of Wnt3a [15], [16] and that this formulation, liposomal Wnt3a (L-Wnt3a), accelerates bone repair [14], [17]. The affinity of Wnt3a for the liposome, the stability of this association, and the means by which L-Wnt3a amplifies endogenous Wnt signaling, were unknown and all are essential parameters in the development of a therapeutic protein. The recent report of the crystal structure of Xenopus Wnt8 (XWnt8) in a complex with its receptor, Frizzled [18] prompted us to characterize this interaction between lipidated Wnt3a and the liposomal bilayer, which generates an unexpectedly stable protein formulation. Using primary cell lines, we assess the kinetics and dynamics of Wnt pathway activation by L-Wnt3a. We then use this information to show that a single, short exposure to L-Wnt3a is sufficient to amplify Wnt signaling in cells for an extended period of time, and therefore significantly improves bone marrow engraftment into a skeletal defect.

## Results

### CHAPS is required to maintain Wnt3a in an active conformation

**Figure 1:**
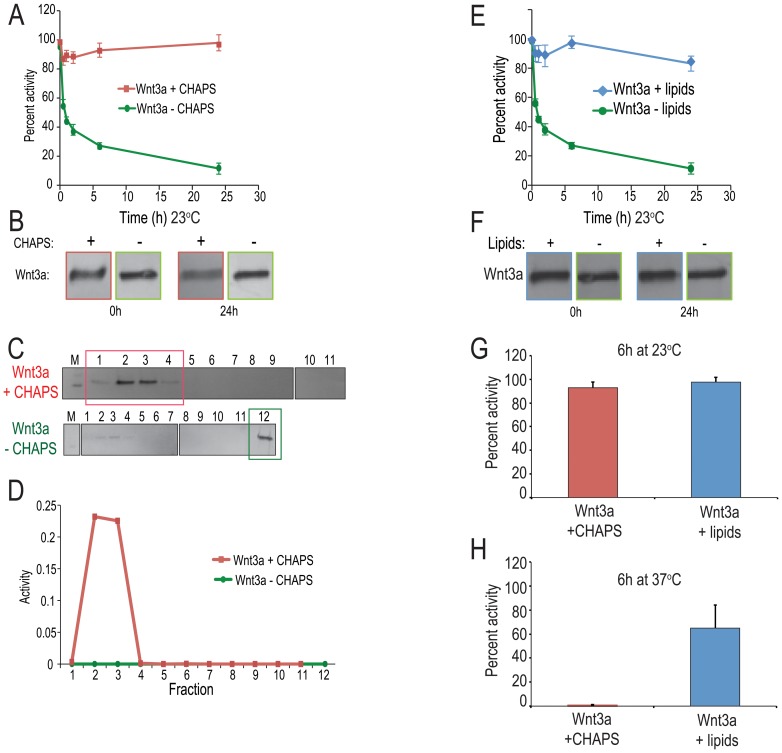
Wnt3a requires a hydrophobic carrier to maintain its biological activity. To generate “Wnt3a+CHAPS” (red), 30 ng/µL of Wnt3a protein was diluted in a buffer containing 1× PBS, 0.5 M NaCl, and 1% CHAPS yielding a final CHAPS concentration of 1% and final Wnt3a concentration of 1.95 ng/µL. To generate “Wnt3a−CHAPS” (green), 30 ng/µL Wnt3a protein was diluted in a buffer containing 1× PBS and 0.5 M NaCl yielding a final CHAPS concentration of 0.004% and final Wnt3a concentration of 1.95 ng/µL. Aliquots of each solution were incubated at 23°C for the indicated times, then applied to LSL cells (see Methods). The output of the LSL assay is luciferase activity/beta galactosidase activity (i.e., luc/lac); percent activity is defined as % activity = 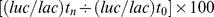, where 100% activity = 0.05 ng/µL Wnt3a. (A) After 24 h at room temperature, Wnt3a+CHAPS retains of its 98.0% activity (red line) whereas Wnt3a−CHAPS (green line) retains 11.4% of its activity. (B) Anti-Wnt3a immunoblotting confirms that equivalent amounts of Wnt3a protein were present in both +CHAPS and −CHAPS conditions at the beginning (0 h) and conclusion (24 h) of the incubation period. (C) Immunoblot analysis confirms that in the presence of CHAPS Wnt co-fractionates with lower density sucrose fractions (#1–3). In the absence of CHAPS the protein precipitates as the majority of the protein is found in a high density fraction (lower panel). (D) Sucrose density gradient centrifugation and Wnt reporter assay, demonstrate that in the presence of CHAPS, Wnt activity (red line) migrate to lower density fractions. Without CHAPS, Wnt activity is not detected (green line) (E) To generate “Wnt3a+lipids” (blue), 30 ng/µL of Wnt3a protein was diluted in DMPC∶cholesterol 90∶10 lipid mixture containing 1× PBS yielding a final lipid concentration of 14 µmoles and final Wnt3a concentration of 1.95 ng/µL. To generate “Wnt3a−lipids” (green), 30 ng/µL Wnt3a protein was diluted in a buffer containing 1× PBS and 0.5 M NaCl yielding a final CHAPS concentration of 0.004% and final Wnt3a concentration of 1.95 ng/µL. Both the samples were incubated at room temperature for 24 h. (F) Anti-Wnt3a immunoblotting confirms that equivalent amounts of Wnt3a protein were present in both +lipids and −lipids conditions at the beginning (0 h) and conclusion (24 h) of the incubation period. Data are mean ±SEM from, or are representative of, at least three independent replicates. (G) Wnt3a incubated in the presence of CHAPS (red bar) and lipids (blue bar) for 6 h at room temperature. Wnt3a samples were prepared as described in (A) and (E). (H) Wnt3a incubated in the presence of CHAPS (red bar) and lipids (blue bar) for 6 h at 37°C.

Wnt3a is post-translationally modified by the attachment of a palmitoleate at Ser209 [4], which renders the protein hydrophobic and unstable in aqueous solutions [3]. Using a Wnt reporter assay [19] we tested whether Wnt3a activity was dependent on the presence of a carrier such as a detergent or lipid vesicle. Wnt3a protein was incubated at 23°C in the presence or absence of CHAPS (3-[(3-cholamidopropyl) dimethylammonio]-1-propanesulfonate) for time periods ranging from 1 min to 24 h, then tested for activity using the Wnt reporter, LSL assay. At room temperature, Wnt3a retained its activity for 24 h provided CHAPS was present (red line, Fig. 1A). Without CHAPS, however, Wnt3a lost activity: after 30 min at 23°C, Wnt3a retained only 56% of its activity (green line, Fig. 1A).

Western analyses at the beginning of the experiment demonstrated that the same concentration of protein was added to the +CHAPS and −CHAPS conditions (Fig. 1B). Therefore, variances in Wnt activity were not due to differences in the amount of Wnt3a added at the outset of the experiment. Western analyses at the 24 h time point showed that the majority of the protein was present as a single band (Fig. 1B). Therefore, the loss of Wnt activity observed in Wnt3a−CHAPS condition was unlikely due to proteolysis.

We hypothesized that in the absence of CHAPS, the hydrophobic Wnt3a protein precipitated out of solution. To test this hypothesis we fractionated Wnt3a+CHAPS and Wnt3a−CHAPS solutions over sucrose gradients, which separate the protein based on density. Each fraction was tested for activity using the LSL assay, and Western blot analyses were used to identify which of the fractions contained Wnt3a.

In the Wnt3a+CHAPS condition, immunoblotting demonstrated that the majority of the protein was restricted to low-density fractions #2 and #3 (Fig. 1C, red box). The LSL activity assay confirmed that these same fractions contained active protein (Fig. 1D, red line). In the Wnt3a−CHAPS condition, immunoblotting demonstrated that the majority of the protein shifted from the low-density fractions to a high-density fraction, #12 which is equivalent to fraction #11 in Wnt3a+CHAPS sample (Fig. 1C, green box). The activity assay showed that fraction 12 contained no active protein (Fig. 1D, green line). Collectively, these experiments indicate that Wnt3a requires CHAPS and without it the protein precipitates and loses activity.

### Lipids can substitute for CHAPS to maintain Wnt3a in an active conformation

CHAPS lyses cell membranes [20] and in our reporter assay, only concentrations below 0.25% of CHAPS was tolerated without significant cell death (Fig. S1A). The same is true in vivo; CHAPS must be eliminated from solutions that are delivered to tissues. We found that DMPC (1,2-Dimyristoyl-sn-Glycero-3-Phosphocholine) and cholesterol lipids could substitute for CHAPS to maintain Wnt3a activity. Recombinant Wnt3a protein was incubated at 23°C in the presence or absence of DMPC∶cholesterol (in a 90∶10 molar ratio) for time periods ranging from 1 min to 24 h and then tested for activity using the LSL assay.

At 23°C, Wnt3a retained 83% of its activity for 24 h, provided DMPC∶cholesterol lipids were present (blue line, Fig. 1E). Without DMPC∶cholesterol, however, Wnt3a lost activity (green line, Fig. 1E). As before, Western blot analyses demonstrated that the same concentration of Wnt3a protein was present in the +lipids and −lipids conditions at the beginning and the end of the experiments (Fig. 1F) indicating that the variance in Wnt activity was not due to differences in the amount of Wnt3a added to the conditions, nor due to proteolysis of the protein during the 23°C incubation period (Fig. 1F). These data demonstrate that DMPC∶cholesterol lipids could stabilize Wnt3a at 23°C, without a measurable loss in activity over the 24 h test period.

### A hydrophobic carrier can stabilize Wnt3a

Thus far our data demonstrate that when a hydrophobic carrier is missing, Wnt3a is unstable and the protein loses activity. However, the stability afforded by CHAPS is temperature-sensitive. For example, at 22°C the critical micellar concentration (CMC) of CHAPS is 4.6 mM but at 36°C, the CMC is 7.1 [21]. Thus, a higher concentration of CHAPS would be required to maintain intact micellar structure but a higher concentration of CHAPS is toxic to cells. This temperature-dependency of CHAPS directly impacted Wnt3a stability: we found that after a 6 h incubation of Wnt3a+CHAPS at 23°C, the protein retained 93% of its activity (Fig. 1G, red bar). After a 6 h incubation of Wnt3a+CHAPS at 37°C, the protein retained none of its activity (Fig. 1H, red bar). Since our goal was to develop Wnt3a as a protein therapeutic, its activity at 37°C was essential. We therefore tested whether lipids were sufficient to stabilize Wnt3a at physiologically relevant temperatures. After a 6 h incubation of Wnt3a+lipids at 23°C, the protein retained 93% of its activity (Fig. 1G, blue bar). After a 6 h incubation of Wnt3a+lipids at 37°C, the protein retained 65% of its activity (Fig. 1H, blue bar). From these experiments we concluded that lipids maintain Wnt3a conformation in an active state. Because lipids are well tolerated by cells, and have been used for in vivo delivery of DNA and small molecules [22], we proceeded to investigate in more detail the interaction between Wnt3a and the lipids.

### Wnt3a stably associates with liposomes

**Figure 2:**
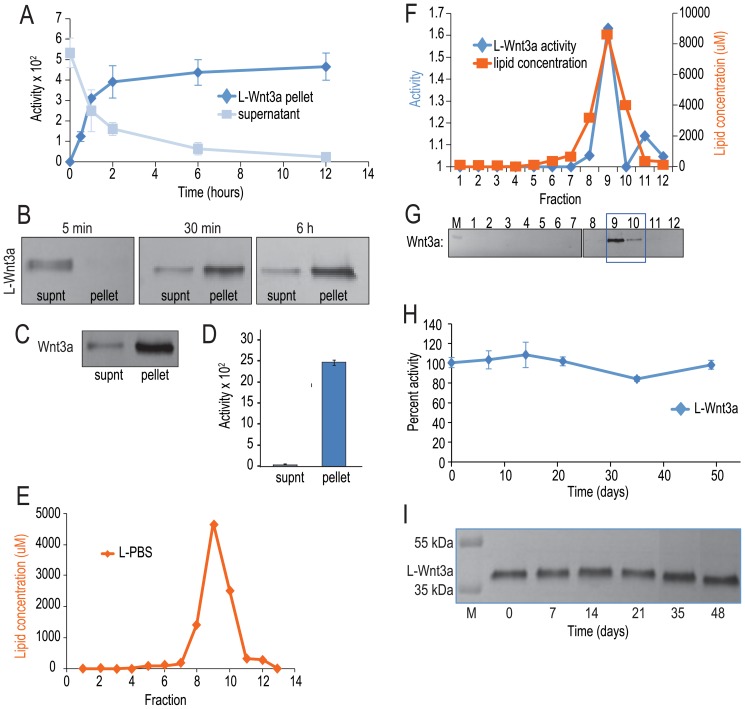
Wnt3a associates with the liposomal surface. (A) The rate of Wnt activity partitioning into the liposomal pellet is shown; initially, all Wnt activity (blue lines) is found in the supernatant but within 30 min, the majority of Wnt activity is associated with the liposomal pellet and by 6 h, 90% of Wnt activity is found in the liposomal pellet. (B) Immunoblot analysis using Wnt3a antibody shows that similar to the Wnt activity, initially the majority of the Wnt is found in the supernatant (supnt); within 30 min majority of the majority of Wnt3a segregates into the liposomal pellet and by 6 h 90% of the protein is associated with the liposomal pellet. (C) Following ultra-centrifugation, the majority of the protein is found in the liposomal pellet. (D) Wnt activity (blue bar) is also found in the pellet. Although some Wnt3a is found in the aqueous supernatant, it is inactive (figure C). (E) Sucrose density gradient centrifugation and phospholipid quantification assay (orange line), demonstrate that PBS liposomes migrate to higher density fractions. (F) Sucrose density gradient centrifugation, phosphatidyl choline lipid quantification and Wnt reporter assay, demonstrate that Wnt activity (blue line), the lipids (orange line), co-fractionate on a sucrose density gradient. (G) Immunoblotting analyses using Wnt3a antibody show that Wnt co-migrates with fractions showing maximum Wnt activity and lipid concentration. (H) The stability of L-Wnt3a is measured. At 4°C, L-Wnt3a retains >80% of its activity after extended storage. (I) An anti-Wnt3a immunoblot reveals no evidence of degradation products of L-Wnt3a after extended storage. Data are mean ±SEM from, or are representative of, at least three independent replicates.

We manufactured DMPC∶cholesterol (90∶10) liposomes as described in the methods (Fig. S1B,C), then incubated the liposomes with Wnt3a at 23°C for varying periods of time (indicated on the X axis, Fig. 2A) and centrifuged to separate the liposomal pellet from the aqueous supernatant. The kinetics of Wnt3a association with the liposomes was then characterized.

Initially (at the 0 h timepoint), all Wnt activity (Fig. 2A) and all Wnt3a protein (Fig. 2B) were in the supernatant. Over the next 30 min, Wnt activity (Fig. 2A) and Wnt3a protein (Fig. 2B) transferred from the supernatant to the pellet at a rate of 3⋅10^−3^ nM/sec (Fig. 2A, B). By 6 h, 90% of the Wnt activity and 90% of the protein were found in the pellet (Fig. 2A, B). After a 24 h incubation at 23°C, liposomes were separated from the aqueous supernatant by centrifugation. No visible protein precipitation was observed in the pellet. Western blot again demonstrated that the majority of Wnt3a was in the liposomal pellet (Fig. 2C). The LSL assay demonstrated that the Wnt activity also localized to the pellet (Fig. 2D). The supernatant contained a minor amount of protein (Fig. 2C), which was inactive (Fig. 2D). From these experiments we concluded that Wnt3a rapidly associates with the liposome and this association is responsible for maintaining the protein's activity.

We investigated the nature of the liposome-Wnt3a interaction. For example, the protein could form an aggregate- rather than an association- with the liposomes. To address this question the protein and liposomes were incubated at 23°C for 6 h to generate liposomal Wnt3a, and then we applied the L-Wnt3a to a sucrose gradient. The LSL assay was employed to pinpoint the fractions containing active Wnt3a and Western blots were used to identify the protein within the various fractions. A phospholipid assay was utilized to detect fractions containing lipids/liposomes (see Methods for details).

As a control we ran PBS-containing (empty) DMPC∶cholesterol 90∶10 liposomes (i.e. L-PBS). The phospholipid assay demonstrated that L-PBS localized to a single, high-density fraction, #9 (Fig. 2E). This showed that L-PBS (Fig. 2E) and Wnt3a protein (Fig. 1C) have distinctly different densities. We then applied L-Wnt3a to a sucrose gradient. Peak Wnt activity localized to fraction #9 (blue line, Fig. 2F). Western analyses demonstrated that the majority of Wnt3a protein also localized to fraction #9 (Fig. 2G). The phospholipid assay demonstrated that fraction #9 contained the majority of the lipids (orange line, Fig. 2F). When compared with CHAPS-solubilized Wnt3a (which segregates in low-density fractions 2–3, see Fig. 1C, D) liposome-associated Wnt3a shifted to a high-density fraction. This shift demonstrated a physical association between Wnt3a protein and the lipids.

The stability of liposome-Wnt3a interaction was tested. Liposomal Wnt3a (L-Wnt3a) was incubated at 4°C for extended periods of time then tested in the LSL assay for activity (Fig. 2H). L-Wnt3a showed no loss of activity over the 48-day test period (Fig. 2H). Western blot analyses indicated no change in amount of protein (Fig. 2I). In a separate 106-day storage period, L-Wnt3a continued to show no loss in activity (not shown). Thus, the physical association between Wnt3a and the liposomal membrane is stable, and is sufficient for pathway activation even after protracted storage at 4°C.

### Liposomal packaging extends the half-life of Wnt3a at 37°C

**Figure 3:**
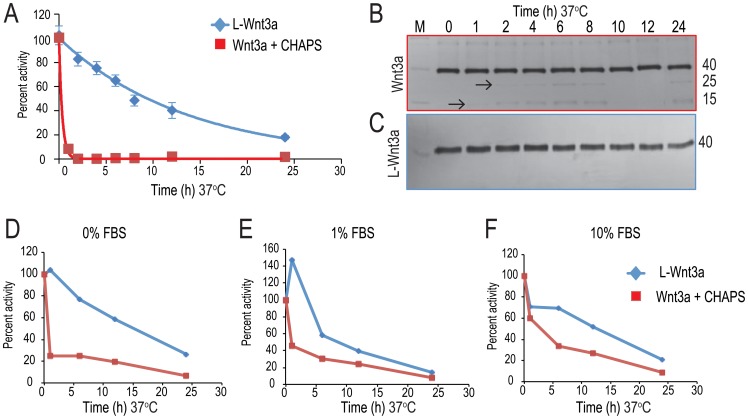
Liposomal packaging improves the stability of Wnt3a protein. (A) L-Wnt3a and Wnt3a (in CHAPS) was incubated at 37°C for 0, 1, 2, 4, 6, 8, 12 and 24 hours. These samples were used to treat LSL cells (see methods and figure 1). Wnt3a protein rapidly denatures and has a half-life of <1 h (red line). At the same temperature, L-Wnt3a resists denaturation and exhibits a half life of ∼10 h (blue line). (B) An immunoblot of these Wnt3a samples incubated at 37°C show smaller molecular weight degradation bands (arrows). (C) An immunoblot showing L-Wnt3a samples incubated at 37°C over the 24 h time course. Data are mean ±SEM from, or are representative of, at least three independent replicates. (D) At 37°C in the absence of serum Wnt3a protein rapidly loses activity (red line) whereas the L-Wnt3a retains activity and has a half-life of 10 h. (E) At 37°C when Wnt3a is incubated in the presence of 1% FBS (red line) loses activity less rapidly than in the absence of FBS and has an half life of 5 h. L-Wnt3a shows similar rate of loss in activity in the presence of 1% FBS as in the absence of FBS. (F) In the presence of 10% FBS, Wnt3a has an improved half life of 10 h (red line) whereas L-Wnt3a (blue line) shows a similar half life in the presence and absence of FBS.

L-Wnt3a is intended for use as a clinical therapeutic, so we tested its stability at 37°C. Data from multiple time points were fit to a single exponential decay, which showed that after 10 h, L-Wnt3a retained half of its activity (Fig. 3A, blue line). In contrast, Wnt3a in CHAPS had already lost half of its activity after only 1 h (Fig. 3A, red line). In Wnt3a+CHAPS samples incubated at 37°C, smaller molecular weight bands were detectable in the immunoblots (Fig. 3B). However immunoblot of L-Wnt3a samples incubated at 37°C did not show the presence of any small molecular weight bands corresponding to Wnt3a (Fig. 3C). Early loss of Wnt activity in Wnt3a+CHAPS solutions was partially attributable to proteolysis, and association with the liposome prevented this proteolysis, thus extending its half-life.

### Serum components stabilize Wnt3a in vitro

Wnt3a protein is a widely used reagent in vitro, where it stimulates both the self-renewal and proliferation of stem and progenitor cell populations [23]–[25]. We wondered about its rapid loss of activity at 37°C in our experiments (Fig. 3A) and hypothesized that hydrophobic components in FBS stabilize Wnt3a activity at 37°C. Therefore we compared the activity of Wnt3a+CHAPS (Fig. 3A) with Wnt3a+CHAPS solutions containing either 1% or 10% FBS. In all these experiments we directly compared the activity of L-Wnt3a with Wnt3a+CHAPS.

Whether L-Wnt3a was incubated in 0%, 1%, or 10% FBS, its activity had a half-life of approximately 10 h (blue lines, Fig. 3D, E, and F). On the other hand, the stability of Wnt3a in CHAPS depended upon the inclusion of FBS. Wnt3a activity in 10% FBS had a half-life of 3 h (Fig. 3F) but as the percentage of FBS was decreased the stability of Wnt3a in CHAPS was completely lost (Fig. 3D,E, red lines). From these data we concluded that Wnt3a requires a hydrophobic carrier (e.g., CHAPS, liposomes, and/or some component of FBS) for stabilization and that at 37°C only lipids keep Wnt3a active for protracted period of time. The clinical use of CHAPS and FBS are contraindicated whereas liposomes are already in use as delivery vehicles. Therefore, we returned to our analyses of L-Wnt3a as a therapeutic protein for in vivo use in humans.

### Mapping the dose response curve for L-Wnt3a in primary cells

**Figure 4:**
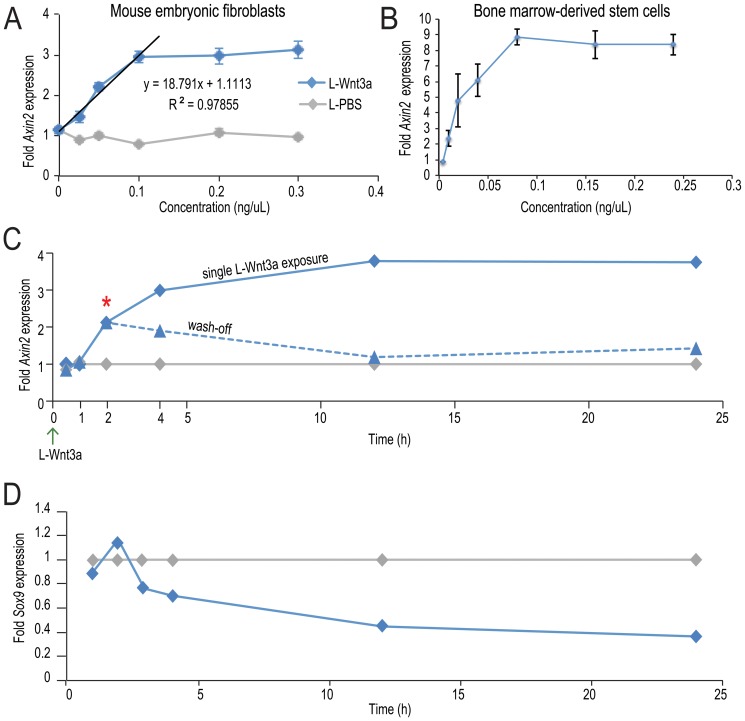
L-Wnt3a activates Wnt/beta-catenin pathway responses in a variety of cell types. Quantitative RT-PCR analyses demonstrate that in (A) primary mouse embryonic fibroblasts and (B) BM-derived mesenchymal stem cells, *Axin2* expression shows a dose-dependent increase in response to increasing L-Wnt3a concentrations. (C) MEFs were incubated with L-PBS (grey line) or L-Wnt3a for varying amounts of time. After 2 h, L-Wnt3a induces *Axin2* expression, just above baseline. After 4 h, L-Wnt3a induced *Axin2* expression has increased 3-fold (blue line). Following a 2 h-incubation with L-Wnt3a the media is removed and incubation continues for varying amounts of time (dashed blue line). 2 h after wash off, *Axin2* expression starts steadily declining and returns to baseline at 12 h. (D) MEFs were incubated with vehicle (grey line) or L-Wnt3a (blue line) for up to 24 h. After 3 h, L-Wnt3a represses *Sox9* expression and keeps it repressed through 24 h of incubation.

Reporter cell lines such as LSL and HEK293T are typically used to assess activity of Wnt proteins and agonists [26]. We validated the activity of L-Wnt3a using both reporter lines (Fig. S2A, B). Under these conditions, both Wnt3a (not shown) and L-Wnt3a showed similar ability to activate Wnt signaling. LSL and HEK293T cells, however, are engineered to be maximally sensitive to Wnt and Wnt agonists and therefore provide little meaningful data on the relationship between dose, drug effect, and clinical response. Therefore, to more closely mimic the in vivo cellular response to a Wnt stimulus, we assayed mouse embryonic limb bud fibroblasts (MEFs) using the expression of the Wnt target gene *Axin2* [27] as a measure of pathway activity. In these primary cells the linear range of effective concentrations was 0.025–0.1 ng/µL Wnt3a (Fig. 4A). We also assayed L-Wnt3a activity in bone marrow-derived stem cells, and found the linear range of 0.004–0.08 ng/µL (Fig. 4B). Thus, these primary cell populations exhibit significantly different sensitivities to Wnt3a stimulation, a finding with direct clinical relevance.

### Determining the temporal dynamics of pathway activation by L-Wnt3a

Having established the dose response range for L-Wnt3a we next evaluated the dynamics of Wnt pathway activation by L-Wnt3a. We used two strategies: in the first case, a single dose of L-Wnt3a was delivered to cells and pathway activation, as determined by *Axin2* expression, was assayed over the next 24 h (Fig. 4C, solid blue line). In the second case, a single dose of L-Wnt3a was delivered to cells and, following a 2 h incubation time, cells were washed and media was replaced; *Axin2* expression as a read out of Wnt signaling was then monitored over the next 22 h (Fig. 4C, dotted blue line). For these analyses we chose to use MEFs; unlike MSCs (mesenchymal stem cells), they can be passaged up to p^4^ and unlike engineered cells, they express negative feedback regulators of the Wnt pathway [28].

Near maximum pathway activation with L-Wnt3a was achieved after 4 h (Fig. 4C). Relative to L-PBS (grey line, Fig. 4C), L-Wnt3a-induced pathway activity remained elevated at the 24 h time point (solid blue line, Fig. 4C). In addition to positively regulated *Axin2*, we also evaluated negatively regulated Wnt target gene, *Sox9* [29]. Within 3 h of treatment, *Sox9* expression is repressed by L-Wnt3a (blue line, Fig. 4D) relative to L-PBS (grey line, Fig. 4D). Thus, by monitoring positively and negatively regulated Wnt targets we show that a single L-Wnt3a exposure results in sustained endogenous Wnt pathway activity.

Pathway activation by L-Wnt3a is reversible. MEFs were incubated with L-Wnt3a for 2 h, which was followed with a wash-off step and continued incubation (dotted blue line, Fig. 4C). *Axin2* expression was evaluated 2, 10, and 22 h after the wash-off. At 2 h (asterisk, Fig. 4C), *Axin2* activation was in the linear range. By 10 h, *Axin2* expression returned to baseline levels (dotted blue line, Fig. 4C). Thus, L-Wnt3a rapidly activates Wnt signaling in MEF's but this activation is transient; once the L-Wnt3a stimulus is removed or degrades, then Wnt signaling returns to baseline.

### L-Wnt3a increases survival and engraftment of bone marrow cells

**Figure 5:**
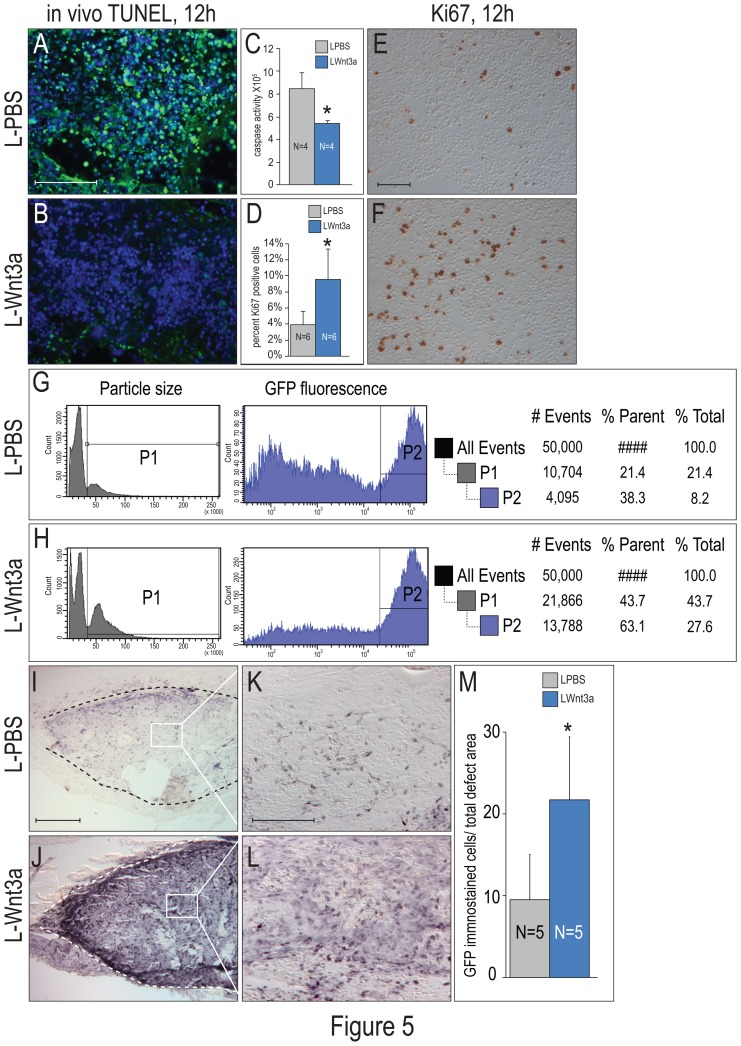
L-Wnt3a stimulates the survival, proliferation, and engraftment of bone marrow cells. (A) Aged BMT were treated with L-PBS or (B) L-Wnt3a with an effective concentration = 150 ng/ml, then transplanted into a skeletal defect and after 12 h, analyzed for DNA fragmentation associated with cell apoptosis using TUNEL. (C) Quantification of caspase activity in aged BM treated with L-PBS (grey bar; N = 4) or L-Wnt3a (blue bar; N = 4). (D) Quantification of Ki67 immunostaining for aged BM treated with (E) L-PBS or (F) L-Wnt3a, then transplanted into a skeletal defect and after 12 h analyzed for cell proliferation. (G) FACS analyses of L-PBS treated BMT, harvested from the defect site on post-transplant day 5. (H) FACS analyses of L-Wnt3a treated BMT, harvested from the defect site on post-transplant day 5. (I) GFP immunostaining identifies the L-PBS treated BMT on post-transplant day 7 (J) GFP immunostaining identifies the L-Wnt3a treated BMT on post-transplant day 7. (K) GFP immunostaining shown in higher magnification demonstrating L-PBS treated BMT on post-transplant day 7 (L) GFP immunostaining shown in higher magnification demonstrating L-Wnt3a treated BMT on post-transplant day 7 (M) Histomorphometric quantification of GFP immunopositive cells in the defect site on post-transplant day 7 (N = 5 for each condition). Abbreviations: GFP: green fluorescent protein; TUNEL: Terminal deoxynucleotidyl transferase dUTP nick end labeling; Scale bars: A,B: 50 µm E,F: 100 µm; I,J: 200 µm; K,L: 50 µm.

Successful bone marrow transplantations (BMT) are contingent on survival and engraftment of the harvested cells [30]. We tested the therapeutic potential of L-Wnt3a to improve engraftment efficiency using an in vivo model of BMT. Whole bone marrow (BM) was harvested then immediately incubated with L-Wnt3a or L-PBS while the recipient site was prepared. During this period we assayed for cellular apoptosis [31] within the graft. Compared to control BMT treated with L-PBS (N = 4; Fig. 5A), BMT treated with L-Wnt3a showed significantly reduced TUNEL staining (N = 4; Fig. 5B). Regulators of apoptosis, of which the caspase family is one of the best-characterized [32], were also assayed. Whole BM was incubated with L-PBS or L-Wnt3a then analyzed after 12 h. L-Wnt3a samples exhibited significantly reduced caspase activity relative to L-PBS treated control groups (N = 4 for each condition; Fig. 5C). L-Wnt3a-mediated cell proliferation was also analyzed. Relative to control, BMT treated with L-Wnt3a showed significantly higher expression of the nuclear protein Ki67 [33] (N = 6 for Fig. 5D; N = 3 for Fig. 5E, F). Thus, a transient exposure to L-Wnt3a promotes both survival and proliferation of cells within the harvested BM.

L-Wnt3a improved the efficiency of BMT. BM harvested from Tg(β-actin-eGFP) donor mice was incubated with L-Wnt3a or L-PBS then transplanted into critical-size skeletal defects in syngeneic mice. Five days after transplantation, cells at the defect site were harvested and FACS was used to isolate grafted GFP^+ve^ cells. This quantitative sorting from three separate experiments revealed that the percent of L-PBS treated GFP^+ve^ cells in the defect site was 38% (Fig. 5G) whereas the percent of L-Wnt3a treated GFP^+ve^ cells in the defect site was 63% (Fig. 5H). Thus, by post-transplant day 5 the number of engrafted cells was measurably higher in the L-Wnt3a treated cases.

We evaluated the BMT two days later, on post-transplant day 7. Here we used GFP immunostaining to identify the transplanted cells (Fig. 5I, J; dotted lines demarcate the transplant). Higher magnification images (Fig. 5K, L) taken of the central region of the transplant (indicated with boxes on Fig. 5I, J) showed that in both L-PBS and L-Wnt3a treated cases (N = 5 for each condition), GFP^+ve^ cells occupied the defect (Fig. 5K, L). Thus, by day 7 the number of engrafted cells had doubled in the L-Wnt3a treated cases (Fig. 5M). Collectively, these data demonstrate that L-Wnt3a treatment improved the survival, proliferation, and the short-term engraftment efficiency of BMT.

## Discussion

The ability to deliver active Wnt proteins with long-term stability, high efficacy, and low toxicity opens a new direction for Wnt-based approaches to regenerative medicine. In previous studies we showed that Wnt3a, when combined with liposomal particles, retains its biological activity in vivo [15], [16]. However the dynamics and kinetics of this association, and the mechanism of pathway activation were unknown. Here, we demonstrate that through its association with the liposome bilayer, Wnt3a becomes unexpectedly stable at physiologically relevant conditions. This stability is due to the unique interaction of the lipidated Wnt3a with the liposomal surface (Fig. 2A, F).

There has been considerable speculation surrounding how Wnt proteins leave the cell membrane and travel to adjacent cells [34]. Drosophila Wingless associates with membrane exo-vesicles (sometimes referred to as argosomes), which is thought to transport Wingless to neighboring cells [35], [36]. Wingless, like almost all Wnt proteins, is lipid modified, and physically associates with lipid vesicles termed exosomes [37], [38].

A number of groups have also suggested that mammalian Wnt proteins are transported between cells via lipid vesicles [38]–[40]. Many studies suggesting that Wnt moves between cells in association with a lipid vesicle rely on gain- and loss-of-function mutations that disrupt essential Wnt chaperone proteins [41], [42]. Using in vitro systems, investigators have shown confocal images showing co-localization of Wingless with the chaperone protein Evi [43] but whether this same transport mechanism is functional in vivo remains to be proven. There is also evidence that once Wnt proteins are associated with the cell surface, their internalization and intracellular signaling takes place inside of endosomal vesicles [44]. Given these findings, liposome-associated Wnt proteins prepared for therapeutic purposes may mimic the vesicle mediated Wnt transport between cells. Panakova and colleagues demonstrated a physical association between Wnt and the exosome membrane [38] and we use a similar approach to demonstrate a similar physical association of Wnt3a with the liposomal membrane. The mechanism by which Wnt associates with the lipid membrane remains unclear. However, one possibility is that Wnt3a is tethered to the membrane via its lipid modification at S209 [4]; another possibility is that it occurs via a charge-charge interaction, and/or via hydrophobic domains on the protein. Clearly, understanding the mechanism whereby Wnt3a associates with the liposomal membrane will provide clues as to how the protein achieves a similar state when being transported between cells in vivo.

We used TEM (transmission electron microscopy) to visualize the range of diameters observed for L-Wnt3a (Fig. S1B, C), which falls within the size range reported for exosomes [45]. The rate of association (Fig. 2A), and the stability afforded to the protein by its association with the liposome (Figs. 2,3) were characterized here but these attributes have yet to be reported for native Wnt proteins. Collectively, these analyses support our conclusion that the liposomal packaging represents a biomimetic strategy for the in vivo delivery of Wnt3a as a therapeutic protein.

Many proteins denature at high temperatures, and avoiding such denaturation at body temperature is key to extending the duration of a protein therapeutic. CHAPS is ineffective in stabilizing the protein at 37°C whereas liposomes are (Fig. 3A,B). Typically, CHAPS stabilizes proteins through its formation of micellar structures that surround the lipid moieties and the hydrophobic domains of the protein; at 37°C, however, this stabilizing effect is lost (Fig. 3) presumably due to the CMC of CHAPS at 37°C. As temperatures are elevated (e.g., from 14°C to 30°C) the degree of hydration of hydrophilic groups decreases, leading to greater repulsion between the polar groups of CHAPS and ultimately, an unfavorable environment for the formation of micelles [46]. Liposomes, on the other hand, are stable at body temperature: The transition temperature of DMPC is 23°C; consequently, at 37°C the liposome membranes are fluid [47], [48]. These fluid liposomal membranes closely resemble cell membranes, which may help to explain why Wnt proteins are extraordinarily stable in a liposomal conformation.

Exogenously applied Wnt proteins have great potential as therapeutics [49] but their safety remains a primary concern. Activating mutations in the Wnt pathway are associated with a wide array of cancers [50], although there is no evidence to date that excessive amounts of exogenously added Wnt ligand itself are problematic. To avoid issues associated with prolonged or uncontrolled Wnt exposure, we opted to test an ex vivo application of L-Wnt3a, where introduction of free Wnt protein into the body is kept to a minimum. Our data indicate that L-Wnt3a can activate the Wnt pathway within 2 h, and pathway activity reaches maximum after 4 h (Fig. 4C); consequently, we targeted bone marrow as a relevant cell population on which to test the therapeutic potential of L-Wnt3a.

Bone marrow (BM) transplants are used for hematopoietic and orthopedic applications. In both clinical applications, a successful outcome depends on survival and engraftment of the transplanted cells and in orthopedic applications, current estimates are that only a fraction of transplanted cells, less than 1%, survive, past the first week [51]. Most strategies to increase the survival and engraftment of BM cells are empirically determined, have met with limited success, and show diminishing efficacy with increasing donor age.

Our data demonstrate that L-Wnt3a incubation improves the survival, proliferation, and ultimately engraftment efficiency of bone marrow cells (Fig. 5). The mechanisms behind this effect are beginning to be elucidated: Bone marrow cells require niche signals for their survival [30], and Wnt proteins are prime candidates for this function (e.g., [52], [53]). We show that L-Wnt3a specifically increases survival of the BM cells by blocking caspase-dependent programmed cell death (Fig. 5). As a consequence, transplanted cells more efficiently compete for host niche microenvironment and give rise to a bony regenerate that forms in the skeletal defect site (Fig. 5).

Questions remain. For example, it remains to be determined whether a brief incubation with L-Wnt3a is sufficient to expand the population of stem cells residing within the whole BM (and thus obviate the need for a second BMT) or whether L-Wnt3a enhances the proliferation of progenitor cells in the graft. Also, safety issues must be addressed, and we must determine whether brief exposure to L-Wnt3a introduces any defects in homing or other functions that are necessary for engraftment. These studies are ongoing.

## Materials and Methods

### Purification of Wnt3a protein

Recombinant mouse and human Wnt3a protein was purified as described previously [3] or was obtained in lyophilized form (R&D Systems). Lyophilized Wnt3a protein was further purified through a 6 kDa MWCO polyacrylamide gel filtration column (Bio-rad) at 4°C. The columns were buffer exchanged with the Wnt3a protein buffer, 1% CHAPS, 0.5 M NaCl, 1× PBS prior to use.

### Generating liposome vesicles

In a 25 mL round bottom flask, 14 µmol of a 90∶10 mol∶mol mixture of DMPC∶cholesterol (Avanti Polar Lipids) was desiccated under a 5 kPa stream of nitrogen gas for 5 minutes, followed by vacuum desiccation at 25**°**C for at least 3 hours. 935 uL of 1× PBS was added to the desiccated lipids and the flask was either sonicated in a water bath sonicator (Branson) for 15 seconds or vortexed for 30 seconds and the reconstituted lipids extruded 35 times through a 100 nm pore-diameter polycarbonate membrane (Whatman GE) at 32°C.

### Preparing liposomal Wnt3a

Purified recombinant Wnt3a protein were incubated with liposome vesicles in a ratio of 13 uL to 187 uL and incubated at 25°C for 6 hours under nitrogen gas. The mixture was ultracentrifuged (Beckman Coulter) at 150×10^3^ g for 1 hour at 4°C to separate the liposome-associated Wnt3a from contaminating BSA. Supernatant was decanted and the L-Wnt3a pellet was re-suspended with an equal volume of 1× PBS then stored under nitrogen gas at 4°C.

### Activity assays

Mouse LSL cells are stably transfected with a Wnt-responsive luciferase reporter plasmid pSuperTOPFlash (Addgene) and a constitutive *LacZ* expression construct pEF/Myc/His/LacZ (Invitrogen) for normalizing beta galactosidase activity to cell number. Human embryonic kidney epithelial (HEK293T) cells are stably transfected with the above two plasmids [15], [16]. Cells (50000 cells/well, 96-well plate) were treated with L-Wnt3a in DMEM supplemented with 10% FBS (Gibco) and 1% P/S (Cellgro) at a concentration of 10 uL in 150 uL total volume, unless otherwise stated. Included also was a serial dilution of purified Wnt3a protein. Cells were incubated overnight at 37°C, 5% CO_2_, then washed, lysed with Lysis Buffer (Applied Biosystems), and the luciferase and β-galactosidase expression levels quantified using a dual-light combined reporter gene assay system (Applied Biosystems). Bioluminescence was quantified with triplicate reads on a dual-light ready luminometer (Berthold).

Activity of L-Wnt3a (ng/uL) or Wnt3a is defined from a standard curve generated by serial dilutions of Wnt3a protein. In experiments involving a time course, Wnt3a activity is expressed as percent activity. Percent activity is calculated as follows:
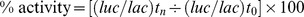



### Immunoblotting

Samples were resolved by SDS-PAGE on a 12% gel and were transferred onto polyvinylidene difluoride membranes (PVDF; Bio-rad). Membranes were incubated with primary antibodies (anti-Wnt3a, R&D Systems) overnight at 4°C and detection was accomplished with secondary antibodies (e.g., anti-rat, IgG Molecular Probes; anti-rabbit IgG, Molecular Probes; anti-mouse IgG, Cell Signaling) conjugated to alkaline phosphatase or horseradish peroxidase. Protein bands were developed with 5-bromo-4-choloro-3-indoyl phosphate (BCIP; Sigma) and nitro blue tetrazolium (NBT; Sigma).

### Sucrose density gradient and PC lipid quantification

500 uL of liposome samples were loaded onto a four step sucrose gradient (1 mL of each of 5%, 10%, 15%, 20% sucrose in 1× PBS, w/v) and centrifuged at 150×10^3^ g for 5 hours at 4°C. Purified Wnt3a was diluted in 1% CHAPS, 0.5 M NaCl, 1× PBS buffer before being loaded onto a four step sucrose gradient that also contained 1% CHAPS. 400 uL fractions were collected starting from the top of the gradient. All fractions were measured for Wnt3a protein activity, total Wnt3a content, and PC lipid content in triplicate reads. PC content was assayed with the EnzyChrom Phospholipid assay kit from BioAssay Systems and quantified by Nanodrop (Thermo).

### Transmission electron microscopy

L-Wnt3a was diluted 100-fold in 1× PBS. 10 uL of diluted L-Wnt3a were placed onto 300 mesh Formvar-coated copper grids (Canemco) and allowed to adsorb for ten minutes. Samples were negatively stained with 2% uranyl acetate for one minute. Samples were observed with an 80 kV JEOL 1400 transmission electron microscope.

### Primary cell harvest and culture

Mouse embryonic fibroblasts (MEFs) were isolated from E10.5 embryos in a CD1 background (Charles Rivers Lab) [29]. Limb buds were amputated, incubated with 0.25% trypsin EDTA (Cellgro) at 37°C for 5 minutes and triturated to dissociate the clumps into single cells. MEFs were cultured using a standard protocol [29]. Briefly, the MEF cell suspension was diluted with media at a ratio of 1∶9 v/v to neutralize the trypsin. Cells were plated in T25 flasks and incubated at 37°C, 5% CO2 for two days with daily media changes. Thereafter cells were expanded into T150 flasks and were cultured for up to p4. MEFs (50000 cells/cm^2^) were treated with L-Wnt3a in DMEM supplemented with 10% FBS. MEFs were incubated for 2 hours at 37°C, 5% CO_2_, unless otherwise stated.

Bone marrow-derived mesenchymal stem cells were harvested from 12 week old wild type mice and cultured as described [54]. Briefly, tibia and femur were cut just below the end of the marrow cavity, longitudinal incisions were made along the shaft of each bone and a 27-guage needle was used to scoop out the fresh marrow. BM cells were cultured in 95 mm culture dishes in 1 ml medium supplemented with FBS and antibiotics at a density of 25×10^6^ cells ml^−1^. Plates were incubated at 37°C with 5% CO_2_. After 8 h the non-adherent cells were removed and the adherent cells were cultured up to p2.

### Bone marrow harvest and transplantation

The Stanford Committee on Animal Research approved all procedures (protocol # 13146). For murine BM harvesting, aged (>10 month old) Tg(β-actin-eGFP) mice (Jackson Lab) were euthanized; femurs and tibiae were collected, split lengthwise and BM was removed with a 21-gauge syringe needle. BM from a single animal was pooled and evenly divided into ∼20 µL aliquots. Each aliquot was incubated in 10 uL of DMEM with 10% FBS to prevent drying. BM was treated with a single dose of 5 uL of either Wnt3a (0.5 ng/uL) or PBS for 4 hours at 37°C. After incubation, BM aliquots were transplanted to skeletal defects (below).

Calvarial skeletal defects were created in aged (>10 month old) syngeneic mice. Mice were anaesthetized with an intraperitoneal injection of Ketamine/Xylazine. An incision was made 3 mm from the sagittal midline to expose the parietal bone. A 2 mm circumferential defect was created using a micro-dissecting trephine attached to a slow speed drill. The pericranium was removed by using a 25-gauge syringe needle and micro-surgery forceps; the dura mater was undisturbed. After transplantation of BM into the defect, the skin was closed using a 6-0 vicryl suture. After surgery, mice received subcutaneous injections of buprenorphine for analgesia until they were euthanized.

### Fluorescence activated cell sorting

FACS was performed on BMT five days after grafting. In brief, host mice were sacrificed and tissue at the injury site was removed using a fluorescent dissection scope to identify the graft. Only GFP^+ve^ tissues were harvested. Cells were recovered from the tissue by collagenase II (0.5%) (DMEM with 1% FBS) digestion at 37°C for 30 min. Every 15 minutes the digest was collected and new digestion solution was added to the remaining tissue. Cells from the first 5 digests were pooled and filtered through a 40 um cell strainer. The resulting cell suspension was analyzed using with Beckton Dickinson fluorescence-activated cell sorter. First, the number of particles was analyzed; only particles corresponding to the size of nucleated cells were counted (e.g., P1). Second, cells were separated based on GFP fluorescence; the P2 population represents GFP^+ve^ cells recovered from the transplant site on day 5.

### Immunostaining

A general procedure is described for immunostaining. In brief, antigen retrieval was performed by incubating slides with Epitope Retrieval Solution (IHCWORD) at 95°C for 20 minutes, or Ficin digestion for 10 minutes at room temperature. Endogenous peroxidase activity was quenched by 3% hydrogen peroxide. Slides were blocked with 5% goat serum and incubated with primary antibodies. Antibodies include rabbit polyclonal anti-GFP (Cell Signaling Technology), and Ki67 (Thermo scientific). Appropriate biotinylated secondary antibodies were used (Vector Laboratories) and signal is either detected using DAB (Vector Laboratories) with the addition of NiCo or Alexa 488 conjugated secondary antibody (Invitrogen) followed by DAPI mounting medium (Vector Laboratories). TUNEL staining was performed as per the manufacturer's instructions (Roche).

### Quantitative PCR

Total RNA was extracted from cell lysates using the RNeasy Miniprep Kit (Qiagen) and cDNA was synthesized from the RNA using a Superscript III First-Strand kit (Invitrogen). PCR was performed on an ABI Prism 7900 HT Sequence Detection System. All reactions were performed in triplicate.

### Statistical analysis

Statistical analyses performed in Microsoft Excel 2007, R v2.13.1, and MATLAB v7.8. Results are expressed as mean ±SEM from the given number of experiments indicated in the figure legends. SEM of variables that are ratios of two random variables were calculated using second order Delta Method approximations and were assumed to be distributed normally.

## Supporting Information

Figure S1 (A) LSL cells stably transfected with TOPflash and LacZ reporter plasmids, were used to demonstrate that CHAPS is toxic to cells. (B) Representative TEM image of an L-Wnt3a preparation and (C) a histogram showing the size distribution of these particles observed under TEM. 968 liposomes from three different liposome preps were counted.
(TIF)

Figure S2 Dose-dependent reporter activity is observed in (A) LSL and (B) HEK293T cell lines following incubation with increasing concentrations of L-Wnt3a.
(TIF)
