# Supplementary figures and images for: Drugging a Stem Cell Compartment Using Wnt3a Protein as a Therapeutic
Source: PLoS One. 2014 Jan 6;9(1):e83650. doi: 10.1371/journal.pone.0083650 (PMC3882211; doi:10.1371/journal.pone.0083650)

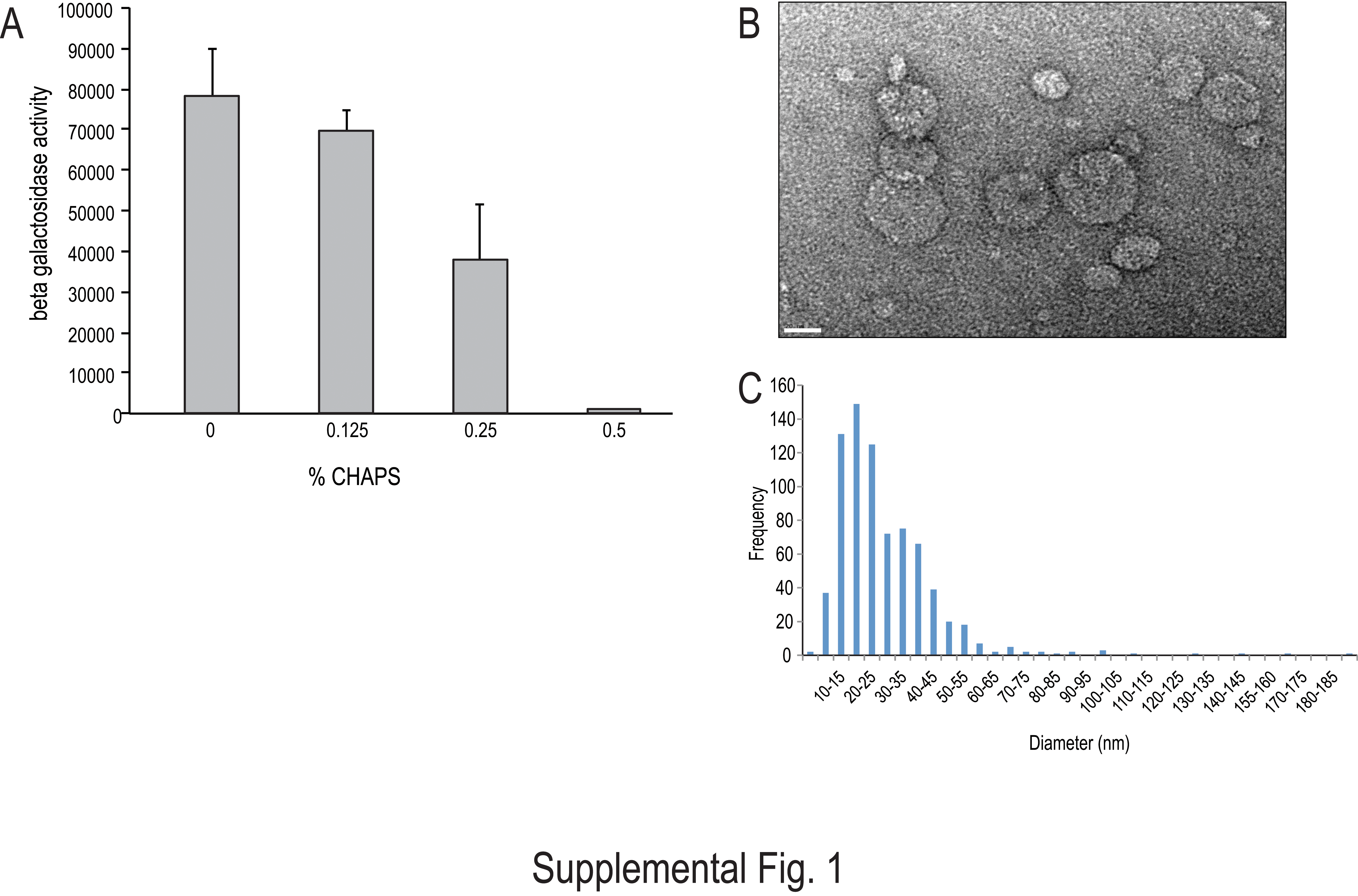

Supplement: Figure S1 — (A) LSL cells stably transfected with TOPflash and LacZ reporter plasmids, were used to demonstrate that CHAPS is toxic to cells. (B) Representative TEM image of an L-Wnt3a preparation and (C) a histogram showing the size distribution of these particles observed under TEM. 968 liposomes from three different liposome preps were counted. (TIF) [file pone.0083650.s001.tif]

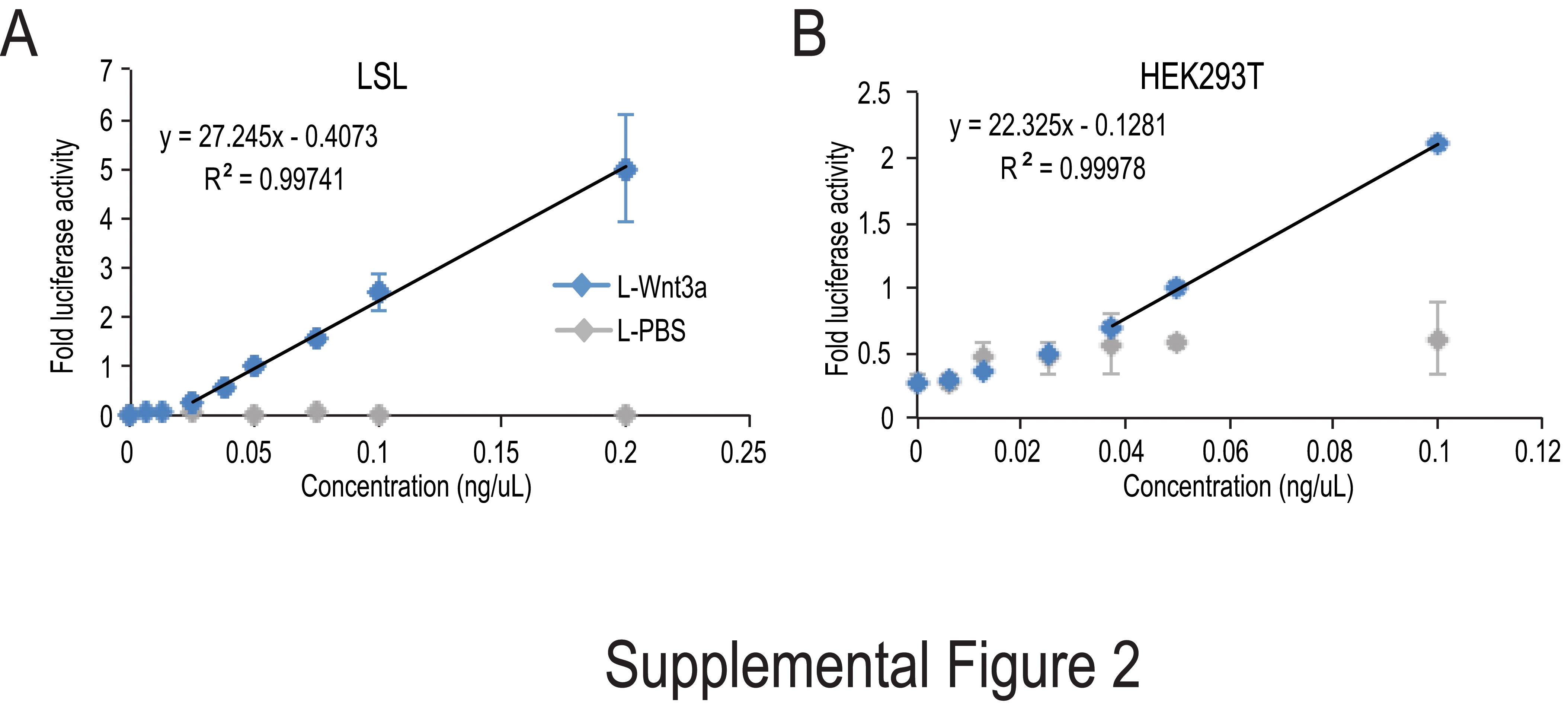

Supplement: Figure S2 — Dose-dependent reporter activity is observed in (A) LSL and (B) HEK293T cell lines following incubation with increasing concentrations of L-Wnt3a. (TIF) [file pone.0083650.s002.tif]
